# Dr. Francis Barker

**Published:** 1860-01

**Authors:** 


DUBLIN has lately lost one of its oldest and most respectable practitioners in the death of DR. FRANCIS BARKER, Professor of Chemistry in Trinity College. He was distinguished for his courtly manners and his sound knowledge, combined with great simplicity of character.

